# Psychosocial Impact of the COVID-19 Pandemic on People With Type 1 Diabetes: Results of an Ecological Momentary Assessment Study

**DOI:** 10.3389/fcdhc.2022.834643

**Published:** 2022-06-20

**Authors:** Fabienne Schmid, Andreas Schmitt, Norbert Hermanns, Bernhard Kulzer, Dominic Ehrmann

**Affiliations:** ^1^ Research Institute Diabetes Academy Mergentheim (FIDAM), Bad Mergentheim, Germany; ^2^ Diabetes Center Mergentheim (DCM), Bad Mergentheim, Germany; ^3^ German Center for Diabetes Research (DZD), Muenchen-Neuherberg, Germany; ^4^ Department of Clinical Psychology and Psychotherapy, University of Bamberg, Bamberg, Germany

**Keywords:** diabetes distress, depression, COVID-19-related burden, ecological momentary assessment, COVID-19 incidence rate

## Abstract

**Aims:**

Psychological distress due to living with diabetes, demanding self-management tasks, impacts on life, and risks of complications is common among people living with diabetes. COVID-19 could pose a new additional risk factor for psychological distress in this group. This study aimed to analyze levels of COVID-19-related burdens and fears, variables explaining these levels, and associations with the concurrent 7-day COVID-19 incidence in people with type 1 diabetes (T1D).

**Methods:**

A total of 113 people with T1D (58% women; age: 42.3 ± 9.9 years) participated in an ecological momentary assessment (EMA) study between December 2020 and March 2021. The participants reported daily levels of COVID-19-related burdens and fears over 10 consecutive days. Global ratings of COVID-19-related burdens and fears were assessed using questionnaires, as were current and previous levels of diabetes distress (PAID), acceptance (DAS), fear of complications (FCQ), depressive symptoms (CES-D), and diabetes self-management (DSMQ). Current levels of diabetes distress and depressive symptoms were compared with pre-pandemic ratings gained during an earlier study phase. Associations between burdens and fears, psychosocial and somatic aspects, and the concurrent 7-day incidence rate were analyzed using multilevel regression.

**Results:**

Diabetes distress and depressive symptoms reported during the pandemic were comparable to pre-pandemic levels (PAID: p = .89; CES-D: p = .38). Daily EMA ratings reflected relatively low mean COVID-19-related burdens and fears in everyday life. However, there was substantial day-to-day variation per person indicating higher burdens on specific days. Multilevel analyses showed that daily COVID-19-related burdens and fears were significantly predicted by pre-pandemic levels of diabetes distress and diabetes acceptance but were not associated with the concurrent 7-day incidence rate nor with demographic and medical variables.

**Conclusions:**

This study observed no increase in diabetes distress and depressive symptoms during the pandemic in people with T1D. The participants reported low to moderate levels of COVID-19-related burdens. COVID-19-related burdens and fears could be explained by pre-pandemic levels of diabetes distress and acceptance but not by demographic and clinical risk variables. The findings suggest that mental factors may constitute stronger predictors of COVID-19-related burdens and fears than objective somatic conditions and risks in middle-aged adults with T1D.

## Introduction

The COVID-19 pandemic is a global health threat on a scale not seen in many years. While any person can be severely affected by the virus, people with pre-existing health problems or chronic conditions are at particularly elevated risk (1). One such risk group is people with type 1 diabetes (2). It has been demonstrated that suboptimal glucose control and pre-existing long-term complications of diabetes increase the risk of a severe clinical course of COVID-19. A recent study found that the odds of hospitalization 14 days after a positive test were 3.9 times higher in people with type 1 diabetes than in comparable persons without diabetes (2). Additionally, people with type 1 diabetes may be at higher risk for infectious diseases, including respiratory tract infections, thus the risk of infection with COVID-19 might also be increased (3). Furthermore, meta-analyses showed a nearly twofold increase in mortality risk for COVID-19-infected people with diabetes vs. without (4, 5).

In addition, adverse effects of the COVID-19 pandemic on psychological well-being and mental health have been observed. Systematic reviews and meta-analyses have shown that between 28% and 34% of people reported increased depressive symptoms due to the pandemic (6, 7). In people with chronic diseases, the prevalence of anxiety and depressive symptoms even be increased to 55% (6). Furthermore, several studies reported increases in mental symptoms in people with diabetes during the pandemic. Fisher et al. (8) found that 67% of people with type 1 diabetes reported higher diabetes distress than before the pandemic. A study by Moradian et al. (9) with German people with type 1 and type 2 diabetes suggests increases in depressive symptoms, anxiety, and psychological distress during the pandemic compared to before, however, using a retrospective evaluation. Moreover, Joensen et al. (10) showed that diabetes distress was positively associated with greater worries about COVID-19 and diabetes in people with type 1 and type 2 diabetes. A study by Brailovskaia et al. (11) demonstrated that depressive symptoms were positively associated with psychological distress caused by the pandemic. Finally, Sauchelli et al. (12) found that the confidence in diabetes self-management decreased during the pandemic and people reported that their needs for assistance and support were not sufficiently met by the diabetes healthcare system.

The psychological repercussions of COVID-19 in people with diabetes are particularly concerning considering the potential effects on diabetes outcomes. Depressive disorders as well as elevated diabetes distress have been frequently associated with detrimental effects on self-care behavior, glycemic control, and quality of life (13). Existing evidence suggests that depression and diabetes distress may have increased during the pandemic. Thus, it is important to understand the psychological impacts and risks that the COVID-19 pandemic poses on people with type 1 diabetes including people’s subjective daily experiences of the pandemic.

We re-examined a sample of people with type 1 diabetes, who had participated in an observational study regarding psychosocial aspects of living with diabetes before the COVID-19-pandemic, during the pandemic. Experiences of burdens and fears due to COVID-19 were captured using questionnaires. Levels of diabetes distress and depressive symptoms as well as fear of complications, acceptance, and self-management were assessed and compared to the pre-pandemic assessment.

Furthermore, we aimed to analyze the subjective experience of COVID-19-related burdens and fears in everyday life. Therefore, we applied ecological momentary assessment (EMA) to assess the day-to-day COVID-19-related experiences. EMA is a methodology allowing the continued daily sampling of participants’ experiences in their everyday life (14).

Finally, we aimed to determine predictors of COVID-19-related burdens and fears, including medical risk factors, psychological aspects, and the concurrent 7-day incidence rate. To our knowledge, this is the first study in people with diabetes that analyzes the associations of the objective risk of infection with COVID-19 (i.e., 7-day incidence rate) with the subjective experience (i.e., burdens and fears) on that day, longitudinally over several days.

## Materials and Methods

The present study was a follow-up of participants of the DIA-LINK Study, a prospective observational study on affective conditions in type 1 diabetes, which was conducted before the COVID-19 pandemic started in Germany. The DIA-LINK study is described in detail elsewhere (14). In short, participants were recruited at a large diabetes clinic in Germany. Participants had to be between 18 and 70 years of age, have type 1 diabetes, and were stratified based on elevated depressive symptoms and diabetes distress levels. Participation in the study went over three months including the baseline assessment, an EMA phase, and a follow-up after three months. This follow-up was used as baseline time point in the current analysis. The study was approved by the Ethics Committee of the German Psychological Society (DGPs) (file number NH082018). The follow-up survey, focusing on participants’ burdens and fears due to the COVID-19 pandemic, which constitutes the basis of the present research, was conducted between December 2020 and March 2021, usually about one year after participation in the original DIA-LINK Study.

### Participant Enrollment

Of the 203 participants of the original DIA-LINK Study, those who had consented to be contacted for a follow-up were informed about the present COVID follow-up *via* email, mail, or telephone. Interested persons were then informed about the follow-up survey, both orally and in writing, and written informed consent was obtained prior to inclusion. A total of 113 former study participants took part in this COVID follow-up. Actual assessment then took place *via* online questionnaires and *via* EMA.

### Assessments

All participants had completed a questionnaire package and interview prior to the beginning of the pandemic as part of their original participation in the DIA-LINK Study. HbA1c had been determined at the same time in a central laboratory from venous blood samples.

In the COVID follow-up, participants were surveyed using EMA over a period of 10 consecutive days. The 10-day period was chosen as it was considered long enough for gaining generalizable results and short enough to avoid participation rejection due to overly high effort. Also, the period should include both week and weekend days to reflect daily patterns of variations. For the EMA, a smartphone app (“mEMA”; Ilumivu Software for Humanity, North Carolina) was installed on the participants’ personal smartphones. Burdens and fears due to the COVID-19 pandemic were assessed each day as part of the evening assessment. A questionnaire survey including a set of questionnaires and specific COVID-19-related questions was administered online at the end of the 10-day EMA period. The most recent HbA1c value was requested personally as part of a telephone interview referring to the most recent estimation as documented in the participants’ diabetes booklets. The following variables were measured before the beginning of the COVID-19 pandemic (baseline):

- Diabetes distress was assessed using the 20-item Problem Areas in Diabetes Scale (PAID) containing diabetes-specific emotional problems and burdens (15). Items are scored on a five-point scale from 0 (“not a problem”) to 4 (“serious problem”). A total score is derived and transformed to a scale from 0 to 100. Scores of 40 and above are considered to indicate elevated diabetes distress (16).
- Depressive symptoms were assessed using the Center for Epidemiologic Studies Depression Scale (CES-D) consisting of 20 items assessing the frequencies of depressive symptoms in the past week (17, 18). Each item is scored on a four-point scale from 0 (“never or rarely”) to 3 (“most of the time”). A total score is calculated ranging from 0 to 60 with higher values indicating higher depressive symptoms. The CES-D has suitable properties in detecting clinical depression (19).
- Fear of diabetes complications was assessed using a short form of the Fear of Complications Questionnaires (FCQ) (20) containing six items. The items request frequencies of complication-related worries or fear and are scored on a four-point scale from 0 (“never or rarely”) to 3 (“most of the time”). A sum score is calculated ranging from 0 to 18 with higher scores indicating higher fear of diabetes complications.
- Diabetes self-management was assessed using the Diabetes Self-Management Questionnaire (DSMQ-R) containing 27 items regarding specific self-management practices (21). Responses are given on a four-point scale from 0 (“does not apply to me“) to 3 (“applies to me very much”). The scale values are transformed to a range between 0 and 10. Higher scores indicate better self-management behavior (21, 22).
- Diabetes acceptance was assessed using a short form of the Diabetes Acceptance Scale (DAS) (23). The scale contains 10 items to be answered on a four-point Likert scale from 0 (“never true for me”) to 3 (“always true for me”). The sum score ranges from 0 to 30 with higher scores indicating higher acceptance of diabetes.

The following variables were measured as part of the assessment during the pandemic (COVID follow-up):

- Participants reported experiences of the COVID-19 pandemic in a self-report scale. Perceived personal burden due to the pandemic, perceived threat, perceived likelihood of infection, and perceived likelihood of a severe course were rated on an 11-point Likert scale from 0 (“very low”/”very unlikely”) to 10 (“very high”/”very likely”).
- Using EMA, the following aspects were assessed once daily during the 10-day period: To determine the impact of the COVID-19 pandemic in everyday life, participants were asked to rate (i) the burden due to worries regarding COVID-19 and health, (ii) the burden due to COVID-19-related restrictions, (iii) the fear of getting infected with COVID-19, and (iv) the fear of family members or friends getting infected with COVID-19. Responses were given on an 11-point Likert scale from 0 (“very low”) to 10 (“very high”).
- Participants also completed the above-mentioned questionnaires (PAID, CES-D, FCQ, DSMQ-R, DAS) again right at the end of the 10-day EMA-period.

### Statistical Analyses

For each of the EMA items, the mean of response scores over the 10 days was calculated for each person (e.g., mean burden level per person). Furthermore, for each item, the mean score across all participants was calculated (e.g., mean burden level in the sample).

In addition, the average course of the EMA item scores over the 10 study days (1–10) was examined. For this purpose, the mean value of each item was calculated for each EMA day (1–10).

To reflect the day-to-day variability of responses of each participant, the coefficient of variation was calculated per person and item. The extent of day-to-day variability across participants is given as mean, median, and 25% and 75% percentiles of the coefficients of variation per item.

To examine possible changes in questionnaire scores, sum scores before and during the pandemic were compared using Student’s *t*-test.

To assess the associations between EMA-based ratings of COVID-19-related burden and the concurrent 7-day incidence rate, multilevel modelling with the participant as the nesting factor was used. Analyses were conducted separately with each EMA item as dependent variable and the 7-day incidence rate on that day as within-level predictor. In the first step, the within factor 7-day incidence rate was entered. In the second step, the medical and demographic risk factors for severe course of COVID-19 were added as between-level predictors, that is, age, sex, BMI, smoking, diabetes duration, presence of diabetes complications, presence of other comorbidities (e.g., cancer), and HbA1c. Finally, in the third step, psychosocial/psychobehavioral predictors were added: diabetes distress (PAID), depressive symptoms (CES-D), diabetes acceptance (DAS), diabetes self-management (DSMQ), and fear of complications (FCQ). The questionnaire scores from before the pandemic were used for the analyses. In each analysis, we controlled for study day and first autoregressive parameter. Bayes estimation was used and raw estimates as well as standardized coefficients (β) are reported.

## Results

### Characteristics of the Study Sample

A total of 113 people with type 1 diabetes participated in the COVID follow-up. The sample characteristics are displayed in **Table 1**. Sixty-six participants (58.4%) were women. The mean age was 43.7 (± 12.0) years. The mean duration of diabetes was 21.6 (± 12.2) years. Fifty-seven persons (50.4%) were diagnosed with at least one long-term complication of diabetes, mostly diabetic neuropathy and/or retinopathy. The mean HbA1c value was 7.8% (± 1.2) or 61.5 (± 13.3) mmol/mol, respectively.

**Table 1:** Characteristics of the study sample (at the time of the pandemic).

Variable	Participants (N = 113)
**Age (years)**	43.7 ± 12.0 (22–70)
**Female sex**	66 (58.4%)
**Smoking**	20 (17.7%)
**BMI (kg/m²)**	27.0 ± 4.9 (18.2–43.9)
**Living alone**	27 (23.9%)
**Persons in household (number)**	2.4 ± 1.1 (1–6)
**Years of education**	13.3 ± 2.4 (9–18)
**Diabetes duration (years)**	21.6 ± 12.2 (2–50)
**With long-term complications** * Retinopathy * Neuropathy * Nephropathy * Foot syndrome * Cardiovascular disease * Arterial vascular disease	29 (25.7%) 40 (35.4%) 4 (3.5%) 2 (1.8%) 1 (0.9%) 4 (3.5%)
**With other serious diseases** * Liver disease * Cancer (past)	6 (5.3%) 2 (1.7%)
**Had severe hypoglycemia requiring assistance in the past year**	15 (13.3%)
**Had ketoacidosis with medical treatment in the past year**	7 (6.2%)
**HbA1c in %** **HbA1c in mmol/mol**	7.8 ± 1.2 (5.5–13.0) 61.5 ± 13.3 (36.6–118.6)
**PAID score (0–100)**	32.4 ± 17.6 (1–71)
**CES-D score (0–60)**	17.9 ± 10.7 (0–44)
**DSMQ score (0–10)**	6.6 ± 1.5 (3.1–9.1)
**FCQ score (0–18)**	8.2 ± 4.3 (0–18)
**DAS score (0–30)**	21.1 ± 7.2 (0–30)
**Perceived burden due to the COVID-19 pandemic (questionnaire item)**	4.99 ± 3.13 (0–10)
**Perceived threat from the COVID-19 (questionnaire item)**	5.04 ± 3.11 (0–10)
**Perceived likelihood of becoming infected later in the pandemic (questionnaire item)**	4.29 ± 2.53 (0–10)
**Perceived risk of severe course if infected (questionnaire item)**	5.00 ± 2.97 (0–10)

Data are M ± SD (range) or n (%). BMI, body mass index; HbA1c, glycated hemoglobin; PAID, Problem Areas in Diabetes questionnaire; CES-D, Center for Epidemiological Studies Depression Scale.

Using the COVID-19-specific questionnaire, the perceived burden due to the COVID-19 pandemic was rated 4.99 ± 3.13 on a scale of 0−10. The perceived threat from COVID-19 was rated with 5.04 ± 3.11 on average. Other risks such as the perceived risk of becoming infected during the pandemic (4.29 ± 2.53) and the risk of severe clinical course if infected (5.00 ± 2.97) were rated similarly (also rated on the questionnaire).

DIA-LINK study participants who did not attend the follow-up survey, compared to those who did (present sample), were significantly younger, more likely to live alone, had higher HbA1c, higher diabetes distress, and more acute complications (i.e., diabetic ketoacidosis) according to baseline assessments (at the time of enrolment) (all *p* ≤.036; data not shown).

### Depression and Diabetes Distress Levels Before and During the Pandemic

**Figure 1:**
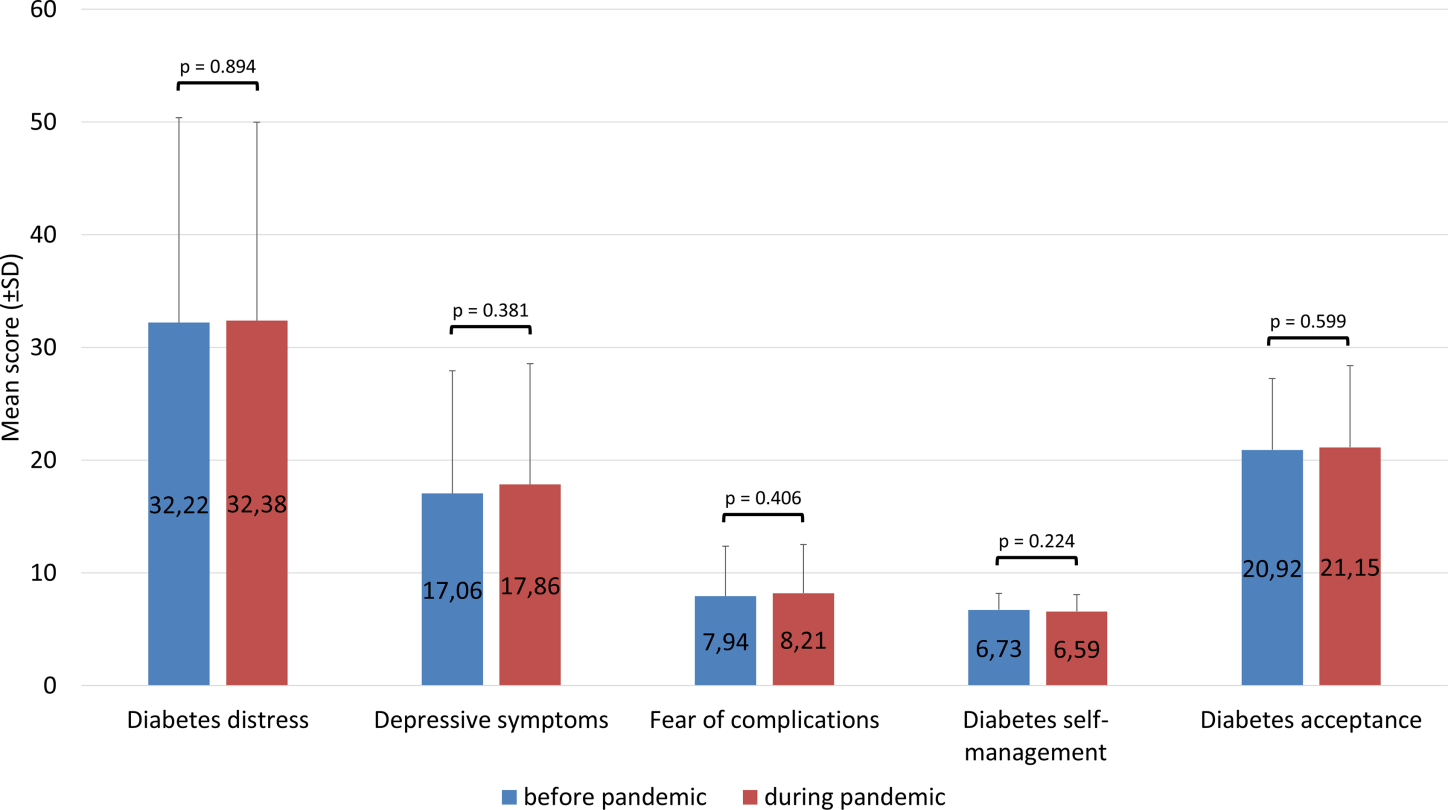
Changes in questionnaire scores before vs. during the COVID-19 pandemic.

**Figure 1** shows the scores of depressive symptoms and diabetes distress before and during the COVID-19 pandemic. Interestingly, neither diabetes distress nor depressive symptoms differed at the time point during the pandemic from the time point before the pandemic (all *p* ≥.38). The mean PAID value was 32.2 ± 18.1 before pandemic and 32.4 ± 17.6 during the pandemic (*p* = .89). The average CES-D score remained stable at 17.1 ± 10.9 before the pandemic and 17.9 ± 10.7 during the pandemic (*p* = .38). In addition, no significant changes were observed for the other questionnaires (**Figure 1**).

### EMA Period: Mean Levels of COVID-19-Related Burdens and Fears

**Figure 2:**
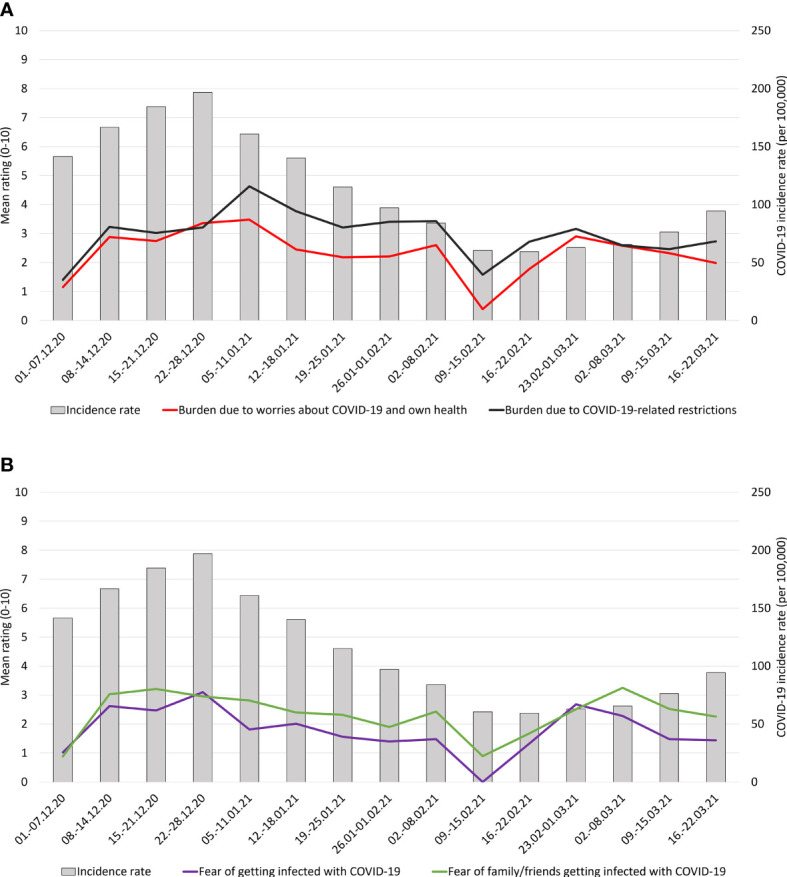
Course of COVID-19-related burdens **(A)** and fears **(B)** displayed against concurrent incidence rates over the study period.

In the daily assessment (EMA), participants reported a mean of 2.3 ± 2.3 (scale: 0−10) regarding burden due to worries about COVID-19 and health. The burden due to COVID-19-related restrictions was rated as 2.9 ± 2.4 on average. The fear of getting infected with COVID-19 was rated with a mean of 1.9 ± 2.0. The fear of family members or friends getting infected with the virus was rated with a mean of 2.3 ± 2.3. **Figure 2** depicts the course of COVID-19-related burdens (**Figure 2A**) and fears (**Figure 2B**) together with the corresponding incidence rates across the study period. Burdens due to worries and restrictions increased toward January 2021 and declined afterward with the nadir in mid-February (**Figure 2A**). Fears of getting infected also showed a slight increase in December 2020 with a steady decline toward March 2021 (**Figure 2B**). Burdens and fears seemed to increase toward April 2021.

### EMA Period: Variability of Burdens and Fears due to COVID-19

The mean day-to-day variability (coefficient of variation) per person of the burden due to worries about COVID-19 and health was 1.14 and indicates that the score varied by 114% around the mean from day to day. Twenty-five percent of individuals had a coefficient of variation of ≤ 0.48 on the question regarding burden due to worries about COVID-19 and health over the 10 days and can be considered relatively stable with respect to their worries. For 25% of all participants, the coefficient of variation was ≥ 1.58, indicating highly fluctuating worry. The coefficient of variation of burden due to COVID-related restrictions was 0.92, indicating that the rating varied by 92% from day to day.

The rating of fear of getting infected with COVID-19 varied from day to day by 114% around the mean. Twenty-five percent of participants had a coefficient of variation ≤ 0.53. In contrast, 25% had a value ≥ 1.38, indicating highly variable anxiety. The mean coefficient of variation of fear of family members or friends getting infected with COVID-19 was 0.97.

**Figure 3:**
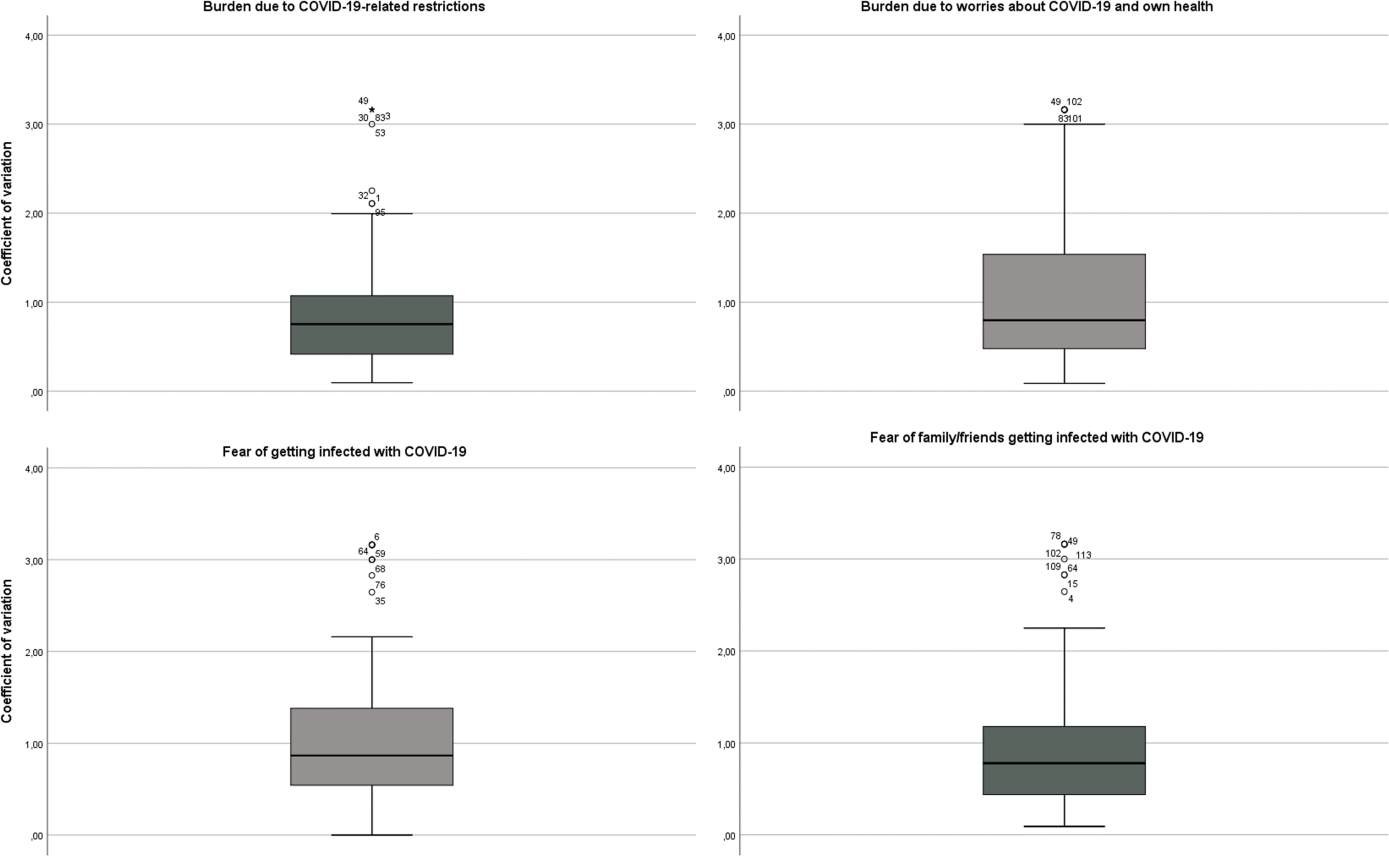
Boxplots displaying variability (CV) of COVID-19-related burdens and fears. Data are bowled line = median; box upper line = 75%; low line = 25%; lower end line = minimum; upper end line = maximum.

Overall, substantial day-to-day variation per person was observed. The results are displayed in **Figure 3**.

### Associations of COVID-19 Burden and Fear Ratings with Risk Factors and the 7-Day Incidence Rate

**Table 2** shows the associations of COVID-19 burdens and fears and 7-day incidence rate. Neither burden due to COVID-19-related restrictions nor burden due to worries about COVID-19 and health, fear of getting infected or the fear of family members/friends getting infected were significantly associated with the concurrent 7-day incidence rate (all *β* < 0.08).

**Table 2:** Multilevel analyses of variables to explain COVID-19-related burdens and fears.

	Burden due to COVID-19-related restrictions		Burden due to worries about COVID-19 and health		Fear of getting infected with COVID-19		Fear of family members or friends getting infected with COVID-19	
Predictor	Estimate (95% CI)	ß	Estimate (95% CI)	ß	Estimate (95% CI)	ß	Estimate (95% CI)	ß
**Step 1 - only within**								
7-day COVID incidence	0.000 (-0.006 - 0.006)	0.01	0.003 (0.000 - 0.006)	0.08	0.002 (-0.002 - 0.008)	0.06	0.002 (-0.004 - 0.009)	0.06
**Step 2 - within + demographic & medical risk factors**								
7-day COVID incidence	0.003 (-0.002 - 0.009)	0.07	0.001 (-0.004 - 0.006)	0.03	0.001 (-0.003 - 0.006)	0.03	0.003 (-0.004 - 0.007)	0.09
Age	-0.021 (-0.060 - 0.021)	-0.10	0.02 (-0.02 - 0.06)	0.11	0.019 (-0.015 - 0.053)	0.11	0.037 (-0.004 - 0.078)	0.19
Female sex	0.542 (-0-323 - 1.388)	0.11	0.517 (-0.348 - 1.330)	0.11	0.244 (-0.502 - 0.990)	0.06	0.803 (-0.063 - 1.705)	0.17
BMI	0.049 (-0.046 - 0.139)	0.10	0.072 (-0.021 - 0.159)	0.14	0.029 (-0.053 - 0.107)	0.07	0.035 (-0.063 - 0.147)	0.08
Smoking	0.498 (-0.878 - 1.909)	0.08	0.969 (-0.302 - 2.326)	0.16	0.838 (-0.241 - 2.066)	0.16	0.765 (-0.370 - 1.972)	0.12
Diabetes duration	0.043 (-0.001 - 0.081)	0.21	0.031 (-0.012 - 0.070)	0.15	**0.040 (0.002 - 0.073)**	**0.22**	**0.040 (0.004 - 0.079)**	**0.21**
With long-term complications	-0.464 (-1.190 - 0.076)	-0.15	-0.289 (-1.014 - 0.255)	-0.10	-0.208 (-0.824 - 0.267)	-0.08	-0.215 (-0.875 - 0.332)	-0.08
With other chronic diseases	1.276 (-.0368 - 3.274)	0.13	0.892 (-0.692 - 2.728)	0.09	0.986 (-0.417 - 2.581)	0.11	1.263 (-0.557 - 2.928)	0.12
HbA_1c_	-0.025 (-0.487 - 0.346)	-0.01	-0.122 (-0.565 - 0.218)	-0.06	-0.135 (-0.508 - 0.173)	-0.08	-0.084 (-0.425 - 0.196)	-0.04
**Step 3 - plus psychosocial risk factors**								
7-day COVID incidence	0.001 (-0.005 - 0.006)	0.02	-0.001 (-0.005 - 0.004)	-0.02	0.000 (-0.004 - 0.004)	0.001	-0.001 (-0.007 - 0.002)	-0.04
Age	-0.022 (-0.067 - 0.024)	-0.09	0.014 (-0.028 - 0.056)	0.06	0.008 (-0.027 - 0.046)	0.04	0.033 (-0.003 - 0.075)	0.15
Female sex	0.484 (-0.481 - 1.383)	0.09	0.271 (-0.602 - 1.105)	0.05	0.084 (-0.674 - 0.801)	0.02	0.465 (-0.348 - 1.263)	0.09
BMI	0.035 (-0.071 - 0.148)	0.06	0.052 (-0.044 - 0.156)	0.09	0.015 (-0.069 - 0.102)	0.03	0.036 (-0.052 - 0.113)	0.07
Smoking	-0.048 (-1.135 - 1.121)	-0.006	0.175 (-0.839 - 1.275)	0.02	0.066 (-0.840 - 0.993)	0.01	0.244 (-0.967 - 1.492)	0.04
Diabetes duration	0.035 (-0.007 - 0.077)	0.14	0.023 (-0.014 - 0.061)	0.09	0.030 (-0.001 - 0.063)	0.15	**0.037 (0.001 - 0.075)**	**0.17**
With long-term complications	-0.432 (-1.083 - 0.152)	-0.13	-0.349 (-0.940 - 0.202)	-0.11	-0.308 (-0.819 - 0.161)	-0.11	-0.276 (-0.771 - 0.268)	-0.09
With other chronic diseases	1.483 (-0.577 - 3.304)	0.12	0.747 (-1.147 - 2.400)	0.06	0.746 (-0.924 - 2.124)	0.08	0.741 (-0.857 - 2.474)	0.07
HbA_1c_	0.066 (-0.317 - 0.416)	0.03	-0.026 (-0.385 - 0.262)	-0.01	-0.029 (-0.352 - 0.233)	-0.01	-0.077 (-0.429 - 0.233)	-0.04
Diabetes distress score (pre-pandemic)	**0.074 (0.026 - 0.113)**	**0.45**	**0.093 (0.050 - 0.127)**	**0.58**	**0.069 (0.032 - 0.099)**	**0.53**	**0.068 (0.033 - 0.100)**	**0.48**
Depressive symptoms score (pre-pandemic)	0.001 (-0.044 - 0.047)	0.004	-0.008 (-0.049 - 0.033)	-0.03	-0.007 (-0.041 - 0.027)	-0.03	-0.004 (-0.046 - 0.039)	-0.02
Diabetes acceptance score (pre-pandemic)	**0.171 (0.067 - 0.264)**	**0.36**	**0.173 (0.080 - 0.255)**	**0.37**	**0.146 (0.065 - 0.219)**	**0.38**	**0.101 (0.010 - 0.190)**	**0.25**
Diabetes self-management score (pre-pandemic)	-0.135 (-0.462 - 0.229)	-0.07	-0.001 (-0.283 - 0.326)	-0.001	0.027 (-0.223 - 0.308)	0.02	0.097 (-0.175 - 0.382)	0.05
Fear of complications score (pre-pandemic)	-0.093 (-0.237 - 0.047)	-0.14	-0.006 (-0.130 - 0.120)	-0.01	0.071 (-0.032 - 0.183)	0.13	0.061 (-0.066 - 0.168)	0.11
**Variation explained by each model *(R*² ´[95% CI])**								
**Step 1 - only within**	0.071 (0.027 - 0.130)		0.092 (0.05 - 0.164)		0.092 (0.045 - 0.155)		0.047 (0.020 - 0.103)	
**Step 2 - within + demographic & medical risk factors**	within: 0.072 (0.029 - 0.128)		within: 0.093 (0.045 - 0.136)		within: 0.091 (0.038 - 0.138)		within: 0.047 (0.016 - 0.092)	
	between: 0.192 (0.062 - 0.343)		between: 0.187 (0.066 - 0.329)		between: 0.189 (0.070 - 0.345)		between: 0.207 (0.092 - 0.362)	
**Step 3 - plus psychosocial risk factors** (pre-pandemic)	within: 0.068 (0.028 - 0.108)		within: 0.095 (0.049 - 0.158)		within: 0.087 (0.043 - 0.139)		within: 0.042 (0.014 - 0.084)	
	between: 0.531 (0.281 - 0.677)		between: 0.606 (0.369 - 0.715)		between: 0.580 (0.351 - 0.697)		between: 0.484 (0.296 - 0.633)	

Data are estimates = unstandardized coefficient (b [95%CI]) or standardized coefficient (ß); BMI= body mass Index, HbA1c = glycated hemoglobin. Significant findings (p < .05) in bold.

The addition of clinical and demographic risk factors in step 2 yielded a slight improvement of explained variation of burdens and fears (**Table 2**). Simply, fear of getting infected as well as fear of family members/friends getting infected were associated with diabetes duration (*β* > 0.21) in this step.

When adding psychosocial risk factors, the explained variation was significantly increased (**Table 2**). Between 48% and 61% of the variation of each aspect could be explained by the models. All COVID-19 items were significantly and positively associated with pre-pandemic levels of diabetes distress (PAID) (all *β* > 0.45) and diabetes acceptance (DAS) (all *β* > 0.25). Higher daily COVID-19-related burdens and fears were significantly predicted by higher diabetes distress before the pandemic. Furthermore, higher daily COVID-19-related burdens and fears were also predicted by higher diabetes acceptance scores notably. In contrast, no demographic or medical variable, except diabetes duration for the fear of infection of family members, was significantly associated with COVID-19-related burdens and fears in the third step.

## Discussion

### Main Findings

The present study found no evidence of increased levels of depressive symptoms and diabetes distress during the COVID-19 pandemic in people with type 1 diabetes. The mean day-to-day ratings of COVID-19-related burdens ranged at a rather low to moderate level. The intra-individual variability of these burdens and concerns were considerable. Elevated diabetes distress and higher diabetes acceptance significantly and independently predicted higher COVID-19-related burdens, whereas the concurrent 7-day incidence rate was not significantly associated.

On average, there was no indication of an increase of diabetes distress and depressive symptoms during the COVID-19 pandemic compared to before in this group of middle-aged adults with type 1 diabetes. This result differs from previous study findings which suggest higher rates of depressive symptoms in the general population (6, 7) as well as higher diabetes distress and depressive symptoms in people with type 1 and type 2 diabetes (8, 9) during the pandemic. On the other hand, the lack of increase in depressive symptoms and diabetes distress is in line with a study by Sacre et al. (24) that also found no increase in people with type 2 diabetes during the pandemic. A possible explanation for the different results in this study compared to Fischer et al. (8) could be the higher mean age of their sample, possibly associated with more COVID-19-related burdens and fears. Furthermore, their study was conducted at an earlier stage of the pandemic at which people with diabetes may have been less habituated to the restrictions and burdens due to COVID-19 (25). Differences to the study by Moradian et al. (9) could be explained by the retrospective evaluation of mental health (depressive symptoms, anxiety, and psychological distress before the pandemic) after the pandemic had begun, which could have overestimated the effect. The lack of change in diabetes distress and depressive symptoms in our study was mirrored by the lack of significant changes in diabetes self-management, fear of complications, and diabetes acceptance notably.

The average daily reported COVID-19-related burdens and fears were lower than those assessed *via* questionnaire. This effect is frequently observed in EMA studies, indicating that questionnaire-assessed burden ratings are usually higher than the day-to-day reported ratings due to more global evaluations and generalization (26, 27).

In the daily assessment over 10 days, the mean levels of burdens and fears were relatively low. However, the individual participant’s burden and fear ratings varied significantly from day-to-day, suggesting that clinically relevant burdens and fears may have been experienced on specific, while not all, days. COVID-19-related burdens and fears showed some level of trend that seemed to follow the daily 7-day incidence rates. However, on a within-person level, there was no evidence of an association of subjective burdens and fears due to COVID-19 and the concurrent objective incidence rate. This analysis showed the benefit of the EMA approach, as objective and subjective risk could be analyzed concurrently daily. Since the burdens and fears were not associated with the 7-day incidence rate in this study, it would be of interest for further research to identify the impacts that lead to greater fluctuations of burdens.

Diabetes distress and diabetes acceptance before the pandemic were the strongest predictors of COVID-19-related burdens and fears. They remained significant even when controlling for more traditional risk factors such as HbA1c and long-term complications. Diabetes distress and acceptance also seemed more relevant for explaining COVID-19-related burdens and fears than the 7-day incidence rate on the respective day. This suggests a partial independence of burdens and fears due to COVID-19 from rather objective risk markers. The finding that higher acceptance of diabetes was related to higher COVID-19-related burdens seems surprising at first look because diabetes acceptance is negatively related with diabetes distress (23, 28). However, considering the objectively higher risks of COVID-19 for people with type 1 diabetes (2), this result may suggest that people who accept their diabetes are also more likely to accept the associated health risks. We hypothesize that low diabetes acceptance, in contrast, might represent rejection and avoidance of dealing with the associated risks for COVID-19. Their perceived personal threat and burden as well as their perceived risk of a severe course if infected might therefore be less pronounced. Further research will be needed to better understand these relations.

The 7-day incidence rate, demographic, and clinical risk factors for COVID-19 infection contributed little to the prediction of COVID-19-specific burdens and fears. It seems that objective risk factors for severe disease progression were less relevant in creating COVID-19-related burdens and fears than psychological aspects such as diabetes-related emotional concerns and integration of diabetes into daily life. Persons reporting higher distress due to their chronic condition also experienced higher burden due to the COVID-19 pandemic. This suggests an overarching way of dealing with stress that can have positive and negative effects, respectively, on both diabetes distress and COVID-19 burden.

### Limitations and Strengths

When interpreting the results, the following limitations must be considered. The conservative findings regarding diabetes distress and depressive symptoms during the pandemic as well as COVID-19-related burdens and fears should be interpreted against the specific characteristics of the study sample, that is, middle-aged adults with type 1 diabetes with relatively good overall health on average. Self-selection may have occurred during recruitment. Compared to the main study, individuals who participated in the follow-up survey had lower HbA1c levels, less diabetes distress, and were less likely to live alone at the time of the original DIA-LINK Study. These are factors that might contribute to lower COVID-19-related burdens and fears. Furthermore, the DIA-LINK Study sample was mainly recruited at a tertiary diabetes center; thus, the sample may not represent people with diabetes in primary care. Comparisons of the changes in diabetes distress and depressive symptoms over time with a control group without diabetes might support a better understanding of the possible impacts, but due to the design of the DIA-LINK Study, controls were not available. Comparisons of the present data with data from the general population would be of great interest; thus, further research will be needed. Finally, the specific time point of the follow-up survey within the pandemic should be considered: the survey was conducted during a period of higher incidence, mainly during the third wave. At that time, lockdown regulations and contact restrictions were in place for the second time in Germany. In addition, the first vaccine against the virus had been approved, which might have led to hopeful expectations. It is unclear to which extent these results can be generalized to other periods, for instance, with lower incidence rates.

Strengths of this study are the assessment of daily impacts of COVID-19 using EMA, probably yielding higher ecological validity than global questionnaire ratings, as well as the direct comparison of depression and diabetes distress levels during the pandemic with pre-pandemic values of the same individuals. Furthermore, fluctuations in COVID-19-related burdens and fears could be made visible *via* EMA demonstrating the additional information compared to single spot questionnaire assessment.

### Conclusions

In summary, the results show substantial day-to-day variability of COVID-19-related burdens and fears in this sample of people with type 1 diabetes. Although the levels of burdens and fears were rather modest on average, clinically relevant levels were experienced on specific days. The findings regarding predictors of COVID-19 burdens and fears suggest that diabetes-specific psychological factors and subjective experiences may be more relevant in explaining burdens and fears than objective health aspects and risk factors for a severe COVID-19 course. The findings highlight the importance of mental factors in dealing with COVID-19 and suggests the need for a psychosocial approach to reducing burdens and worries due to the pandemic in addition to information/education about a person’s individual risk to foster realistic expectations and corresponding feelings.

## Data Availability Statement

The raw data supporting the conclusions of this article will be made available by the authors, without undue reservation. The dataset analyzed for this study is restricted by the German Federal Data Protection Act (BDSG) and will be made available upon reasonable request to the corresponding author.

## Ethics Statement

The studies involving human participants were reviewed and approved by Ethics committee of the German Psychological Society. The patients/participants provided their written informed consent to participate in this study.

## Author Contributions

FS: collected the data; analyzed and interpreted the data; drafted the manuscript. AS: planned and designed the study; collected the data; discussed the findings; revised the manuscript. NH: planned and designed the study; discussed the findings; revised the manuscript. BK: planned and designed the study; discussed the findings. DE: planned and designed the study; analyzed and interpreted the data; revised the manuscript. All authors contributed to the article and approved the submitted version.

## Funding

This work was supported by the German Center for Diabetes Research (DZD) [grant number 82DZD11A02]. The funders were not involved in decisions regarding study design; collection, analysis, and interpretation of data; writing of the report; and submission of the article for publication.

## Conflict of Interest

The authors declare that the research was conducted in the absence of any commercial or financial relationships that could be construed as a potential conflict of interest.

## Publisher’s Note

All claims expressed in this article are solely those of the authors and do not necessarily represent those of their affiliated organizations, or those of the publisher, the editors and the reviewers. Any product that may be evaluated in this article, or claim that may be made by its manufacturer, is not guaranteed or endorsed by the publisher.
